# Muscle protein metabolism in neonatal alloxan-administered rats: effects of continuous and intermittent swimming training

**DOI:** 10.1186/1758-5996-4-5

**Published:** 2012-02-06

**Authors:** Carla Ribeiro, Lucieli T Cambri, Rodrigo A Dalia, Michel B Araújo, Ana C Ghezzi, Leandro P Moura, Gustavo G Araújo, Jose D Botezelli, Maria AR Mello

**Affiliations:** 1 São Paulo State University - UNESP, Physical Education Department, Av: 24-A, 1515 Bela Vista. CEP: 13506-900 Rio Claro - São Paulo - Brazil

**Keywords:** DMT2, exercise training, protein synthesis, hypertrophy muscle

## Abstract

**Background:**

This study aimed to examine the effects of intermittent and continuous swimming training on muscle protein metabolism in neonatal alloxan-administered rats.

**Methods:**

Wistar rats were used and divided into six groups: sedentary alloxan (SA), sedentary control (SC), continuous trained alloxan (CA), intermittent trained alloxan (IA), continuous trained control (CC) and intermittent trained control (IC). Alloxan (250 mg/kg body weight) was injected into newborn rats at 6 days of age. The continuous training protocol consisted of 12 weeks of swimming training in individual cylinder tanks while supporting a load that was 5% of body weight; uninterrupted swimming for 1 h/day, five days a week. The intermittent training protocol consisted of 12 weeks of swimming training in individual cylinder tanks while supporting a load that was 15% of body weight; 30 s of activity interrupted by 30 s of rest for a total of 20 min/day, five days a week.

**Results:**

At 28 days, the alloxan animals displayed higher glycemia after glucose overload than the control animals. No differences in insulinemia among the groups were detected. At 120 days, no differences in serum albumin and total protein among the groups were observed. Compared to the other groups, DNA concentrations were higher in the alloxan animals that were subjected to continuous training, whereas the DNA/protein ratio was higher in the alloxan animals that were subjected to intermittent training.

**Conclusion:**

It was concluded that continuous and intermittent training sessions were effective in altering muscle growth by hyperplasia and hypertrophy, respectively, in alloxan-administered animals.

## 1. Background

Diabetes is a group of metabolic disorders characterized by hyperglycemia resulting from defects in insulin secretion or insulin action [1-3], which leads to changes in carbohydrate, fat and protein metabolism. The chronic hyperglycemia of diabetes is associated with dysfunction and failure of various organs and tissues, including muscle. Type 1 diabetes mellitus (T1DM) is a multifactorial autoimmune disease in which susceptibility is determined by a combination of genetic and environmental factors [2,4]. The disease is characterized by chronic hyperglycemia and by the development of specific vascular alterations [2,5]. In this diabetes type, there is a T cell-mediated destruction of the insulin secreting β-cells of the pancreatic islets. Type 2 diabetes (T2DM) accounts for approximately 90 to 95% of diabetics and may be promoted by several factors, such as obesity, a high calorie diet and physical inactivity [2,6]. T2DM is characterized by a combination of insulin resistance and a compensatory response to inadequate insulin secretion, which leads to the characteristic hyperglycemia [2,7]. Currently, it has been observed that T2DM changes the serum levels of several amino acids that may contribute to disturbances in insulin secretion and action [8].

It is currently accepted that physical training is of great importance in improving the level of physical fitness and quality of life. Exercise has been increasingly recommended in the prevention and rehabilitation of cardiorespiratory diseases, osteoporosis, diabetes, and in combating stress [9-11]. Particularly, in the treatment of T2DM, regular physical exercise at different intensities improves glucose tolerance and reduces insulin resistance. However, the literature lacks direct evidence on the preventive effect of exercise on the development of T2DM; in particular, investigations into the effect of effort intensity and different exercise protocols is lacking because this type of research is more difficult to conduct in humans. In this context, animal models are more suitable for the study of these issues.

Beta-cytotoxic chemical agents have been widely and successfully employed as experimental model for studies of the diabetes-induced complications successfully [12-14] Alloxan is a drug widely used as a diabetes experimental model. This drug, presents an effect selectively toxic on the beta cells from the islets of Langerhans in the pancreas [15]. It is known that the application of diabetogenic drugs in adult animals produces severe diabetes and in many cases requires the use of insulin when rats need to survive for a longer period of time [16], but in newborn animals a few days after the drug administration occurs a decrease in pancreatic insulin production. From this phase on, β cells regenerated spontaneously, by replication and/or neogenesis [17], reaches 50% of the value presented for control animals [18].

Portha et al. [18] described an experimental model of neonatal diabetes in Wistar rats by the application of streptozotocin on the day of birth. In this model, it was demonstrated that hyperglycemia is transitory. The blood glucose levels normalize after the first week of life, with restoration of insulin production and β-cell mass. That study features a T2DM model in rats, in which the experimental animals presented good survival [18]. Takada et al. [19] using the neonatal streptozotocin model - administered in the animals on the 5th day of life, showed that after 12 weeks of the experiment the pancreatic insulin content decreased and impaired glucose tolerance. Moreover, in another study using the streptozotocin application as an experimental model in which it was administrated at 4 days after birth, it was observed, after three weeks of streptozotocin injection, a 72% decrease in pancreatic insulin stores without change in pancreatic glucagon stores [20].

Later, Kodama et al. [21] developed another model that replaced streptozotocin with alloxan. In that study, alloxan was administered on the 2th, 4th or 6th days of life. When analyzed at 60 days of life, the rats that received alloxan on the 2th of life showed blood glucose levels in the fed state that were slightly higher than those of the control rats, whereas the rats that received the drug on the 4th and 6th days showed blood glucose levels that were significantly higher than those of the controls. The authors considered a useful model for studies on chronic complications of diabetes. However, they emphasized that more studies were needed to determine whether the alloxan-administered neonatal rats showed characteristics of T2DM, as was seen in the streptozotocin neonatal model.

Oliveira et al. [22] examined the fasting plasma glucose and glucose tolerance in the male rats at 30, 60, and 90 days old after receiving alloxan on the 2nd day of life. Fasting glucose was not different from control rats at any time. At 90 days the static insulin secretion by the pancreatic islets of the animals was similar among the groups at 2.8 and 5.6 mM of glucose, but the pancreatic insulin content of the alloxan group was smaller than the control group [17]. Contarteze et al. [23] showed that these animals presented higher blood glucose levels after an oral glucose load than controls. Similar results were reported by Cimbiz et al. [3], in which the alloxan group presented higher blood fasting glucose levels than the control group. Ribeiro et al. [24], during a glucose tolerance test in rats that received alloxan on the 6th day of life, also showed higher blood glucose values in the alloxan group compared to the control group at 28 days old and 60 days old. Moreover at 60 days old, it was observed lower insulin secreted by the isolated islets in relation to the control group for the glucose concentration of 16.7 mM. Taken together, these data show that the neonatal alloxan administration model illustrates interesting features for studying the role of exercise in preventing and treating diabetes. Studies using different training protocols in the prevention and treatment of T2DM are scarce especially in relation to protein metabolism.

A study in rats that were subjected to the neonatal application of alloxan and continuous swimming training at moderate intensity showed that the total serum protein and skeletal muscle protein content did not change after 8 weeks of exercise training [22]. Luciano and Mello [25], using the alloxan model of diabetes in adult rats, did not observe any differences in total protein and albumin concentrations between the groups after 4 weeks of moderate continuous swimming training.

In adult alloxan-mediated diabetic rats Leme et al. [26], there were no changes in serum albumin after 8 weeks of continuous swimming exercise at an intensity of 90% the maximal lactate steady state. On the other hand, non-diabetic rats that were subjected to high-intensity intermittent training showed higher expression of GLUT-4 protein in skeletal muscle [27]. Fedele et al. [28] showed that in contrast to non-diabetic animals, partial pancreatectomy diabetic animals did not show increased protein synthesis after acute resistance training. Therefore, it seems that both continuous and intermittent training may play important roles in the prevention and treatment of diabetes, but further studies are needed to characterize the intensity and frequency that are best suited to each of these protocols. In this context, the present study aimed to examine the effects of intermittent and continuous training on muscle protein metabolism in neonatal alloxan-administered rats.

## 2. Methods

### 2.1 Animals

The studies were conducted on newborn male Wistar rats that were kept at 25 ± 1°C under a 12 h/12 h light-dark cycle and fed standard rodent chow and water ad libitum. During breastfeeding, food and water were offered ad libitum to the mothers, and the pups were distributed in litters of eight per dam. All animal experiments were approved by the committee of ethics in animal research, Taubate University-CEEA/UNITAU, under protocol n° 019/08.

### 2.2 Neonatal alloxan administration

At 6 days old, after a 15-h fast, the male pups with a body weight of 11.9 ± 1.2 g received intraperitoneal alloxan monohydrate (Sigma-Aldrich Inc., St Louis, MO, USA) injections (250 mg/kg body weight) that were dissolved in 0.01 M citrate buffer at pH 4.5 [29]. Vehicle-injected (citrate buffer) rats were used as controls. Immediately, the pups were distributed so that each mother nursed eight of them.

### 2.3 Experimental groups

**Figure 1:**
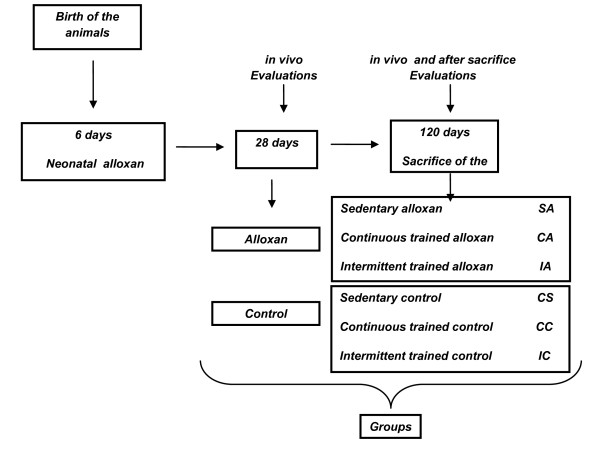
**Experimental Design**.

At 28 days old, the animals were randomly divided into six groups of 10 rats each and remained under observation until 120 days old (Figure 1):

- Sedentary Control (SC): citrate buffer-injected rats that were not subjected to exercise training

- Continuous Training Control (CC): citrate buffer-injected rats that were subjected to the continuous exercise training protocol.

- Intermittent Training Control (IC): citrate buffer-injected rats that were subjected to the intermittent exercise training protocol.

- Sedentary Alloxan (SA): alloxan-injected rats that were not subjected to exercise training.

- Continuous Training Alloxan (CA): alloxan-injected rats that were subjected to the continuous exercise training protocol.

- Intermittent Training Alloxan (IA): alloxan-injected rats that were subjected to the intermittent exercise training protocol.

### 2.4 Exercise training

The rats were initially adapted to the water to reduce the stress caused by the physical exercise performed in this environment. The adaptation consisted of the following sequence, which was followed by the initiation of training: 5, 10, and 15 min in shallow water; 5, 10, and 15 min in deep water; 5 min with a bag tied to the thorax; and 5, 10, and 15 min with a bag containing a load of 3% of body weight tied to the thorax. Between weaning and 120 days of age, the animals that were trained with the continuous exercise protocol were subjected to 1 hour per day, five days a week, of uninterrupted swimming in individual cylinder tanks (25 cm in diameter × 50 cm in depth) while supporting a load of 5% of body weight. This intensity corresponds to the aerobic/anaerobic metabolic transition during swimming exercise for rats [30]. Between weaning and 120 days of age, the animals that were trained with the intermittent protocol were subjected to 5 days a week of swimming in individual cylinder tanks (25 cm in diameter × 50 cm in depth) for 30 s interrupted by 30 s of rest for a total of 20 min per day while supporting a load of 15% of body weight (adapted from [31]). The water temperature was maintained at 31 ± 1°C, which was considered a thermally neutral temperature for rats [32,33]. The training protocols consisted of equivalent total weekly training load (WL). According to Araújo et al. [34], WL represents the total of stimuli of training obtained by the product of the exercise time (t) and the intensity (%). Therefore, in this study, the continuous training protocol, in which WL = 60 min × 5% = 300, was equivalent to the intermittent training protocol, in which WL = 20 min × 15% = 300.

### 2.5 In vivo Evaluations

The body weight, food and water intake of all the animals were registered once a week from weaning on. They were also evaluated for fasting and non-fasting blood glucose and insulin levels.

#### 2.5.1 Blood glucose and insulin

The diabetogenic effect of alloxan was confirmed when the rats were 28 and 120 days old by the determination of the fasting (after a 12-h fast) and non-fasting (30 min after administration of glucose solution, 2.0 g [kg body weight]-1, into the stomach through a gastric catheter) serum glucose by the oxidase method (Kit glicose - Laborlab: CAT n° 02200- Guarulhos- SP) and the serum insulin based on ELISA (Kit Insulina- Diagnostic Systems Laboratories INC (DSL), REF: 10-1600, 445 medical Center BLVD, Webster, TX 77598, USA).

### 2.6. Biological material collection

At 120 days of age, biological material was obtained from animals that were sacrificed by decapitation after deep anesthesia with sodium amobarbital (15 mg/kg body weight) without fasting and 48 hours after the last in vivo procedure.

#### 2.6.1. Blood

Blood samples were collected to verify the concentrations of glucose (Kit glicose - Laborlab: CAT n° 02200- Guarulhos- SP), total protein and albumin by spectrometry (total protein Kit - Laborlab: CAT n° 03801 e albumin Kit - Laborlab: CAT n° 03802- Guarulhos- SP) and serum insulin by ELISA (Kit Insulina-Diagnostic systems laboratories INC (DSL), REF: 10-1600, 445 medical Center BLVD, Webster, TX 77598, USA).

#### 2.6.2. Adipose Tissue

The adipose tissue of the posterior subcutaneous, mesenteric and retroperitoneal regions were removed and weighed for determination of total fat content. Excision of the different fat deposits was carried out according to the description of Cinti [35]

#### 2.6.3. Gastrocnemius muscle

Samples of the gastrocnemius muscle were excised for the evaluation of total protein [36] and DNA content [37], to determine the number (DNA) and size (protein/DNA ratio) of the cells [38].

#### 2.6.4. Soleus muscle

##### 2.6.4.1 Protein Synthesis

Longitudinal strips from the soleus muscle (70 mg) were preincubated for 30 min in a RPMI 1640 medium (with glutamine and without red phenol and sodium bicarbonate) that was supplemented with fat-free 0.1% bovine serum albumin (BSA) and 100 μU ml-1 insulin and saturated with a 95% O2/5% CO2 gas mixture. The strips were then transferred to fresh RPMI medium, which contained the same supplements in addition to 0.05 ACi ml-1 [14C] phenylalanine, and incubated for two more hours. At the end of this incubation, the muscle strips were homogenized in 5% trichloroacetic acid (TCA) and centrifuged at 2000 rpm for 15 min at 4°C. TCA-insoluble material was washed three times with 5% TCA. The resulting precipitate was dissolved for 30 min in 10% sodium dodecyl sulfate at room temperature to determine both protein content and the radioactivity incorporated into the muscle protein. Muscle protein content was determined by the folin phenol method [38], and the radioactivity incorporated into the muscle proteins was determined with a liquid scintillation counter. Protein synthesis was calculated by dividing the incorporated radioactivity by the specific phenylalanine radioactivity in the incubation medium.

##### 2.6.4.2 Protein Degradation

Tyrosine liberation by isolated muscles in the presence of cyclohexamide was employed as the protein degradation index [39]. This method is based on the fact that the amino acid tyrosine is neither synthesized nor degraded by skeletal muscle. Longitudinal strips from the soleus muscle (70 mg) were preincubated in Krebs-Ringer buffer (NaCl, 1.2 mmol/L; KCl, 4.8 mmol/L; NaHCO3, 25 mmol/l; CaCl2, 2.5 mmol/L; KH2PO4, 1.2 mmol/l; MgSO4, 1.2 mmol/L; pH 7.4) that was supplemented with 5.5 mmol/L glucose, 1.34% BSA, 5 AUml-1 insulin, and 5.0 mmol/L cyclohexamide and saturated with a 95% O2/5% CO2 gas mixture. Subsequently, the muscle strips were transferred to fresh medium with the same composition and incubated for two more hours. At the end of the incubation, the samples of the incubation medium were used for tyrosine determination [40].

#### 2.6.5. Pancreatic Islets

To measure insulin secretion, groups of five pancreatic islets isolated by collagenase digestion [41] were incubated for 30 min at 37°C, in Krebs bicarbonate medium containing glucose (5,6 mM), supplemented with bovine serum albumin (3 g/l) and balanced with a mixture of 95% O_2 _-5% CO_2; _pH 7,4. The solution was then replaced by fresh buffer, and the islets were incubated for a further hour with different glucose concentrations (2.8, 11.1 mM). The insulin content of the incubation medium was determined by ELISA (Kit Insulina- Diagnostic Systems Laboratories INC (DSL), REF: 10-1600, 445 medical Center BLVD, Webster, TX 77598, USA).

### 2.7. Statistics

Data analysis was performed using the student t-test or two-way analysis of variance ANOVA followed by Bonferroni post hoc test when appropriate. In all cases, the level of significance was determined at 5% (p < 0.05).

## 3. Results

**Figure 2:**
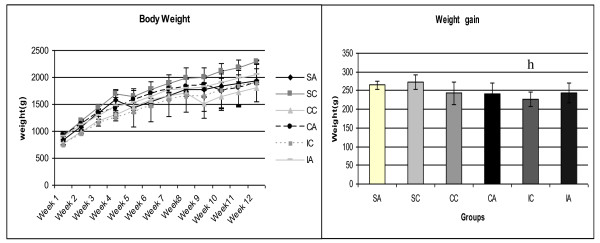
**Body weight and weight gain of the animals at weaning (28 days) until the end of the experiment (120 days)**. Results are expressed as means ± standard deviation of 10 animals per group. SA: Sedentary alloxan, SC: Sedentary control, CC: Continuous training control, CA: Continuous training alloxan, IC: Intermittent training control and IA: Intermittent training alloxan. Letter indicate a statistically significant difference among groups (Two- way ANOVA (3 × 2) and Bonferroni post hoc test, p < 0.05) referring to weight gain. h, statistically significant difference for SA vs. IC.

**Figure 3:**
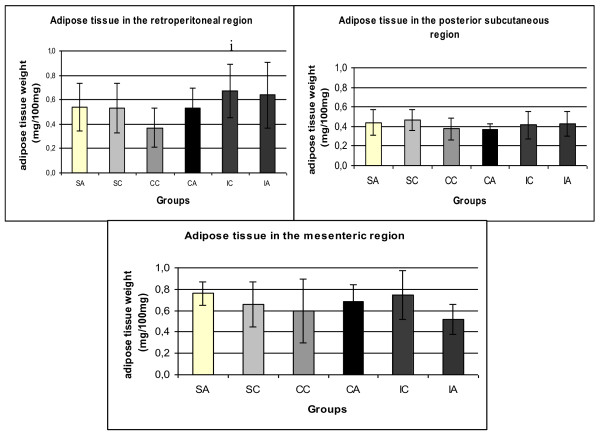
**Adipose tissue weight (mg/100 mg) of the retroperitoneal, posterior subcutaneous and mesenteric regions**. Results are expressed as means ± standard deviation of 10 animals per group. SA: Sedentary alloxan. SC: Sedentary control. CC: Continuous training control. CA: Continuous training alloxan. IC: Intermittent training control and IA: Intermittent training alloxan. Letter indicate a statistically significant difference among groups (Two- way ANOVA (3 × 2) and Bonferroni post hoc test. p < 0.05) referring to non fasting serum glucose. i. statistically significant difference for CC vs IC.

Changes in animal body weight (Figure 2A) during the experimental period were analyzed by the weight gain during the experiment, shown in Figure 2B. The IC group had reduced weight gain compared to the SC group, which demonstrates the efficacy of intermittent training in weight control in the control animals. The adipose tissue weight of the retroperitoneal, posterior subcutaneous and mesenteric regions were presented in Figure 3A, B and Figure 3C, respectively. It was observed significant difference only in the retroperitoneal fat content in which the CC group showed significantly lower values than the IC group.

**Figure 4:**
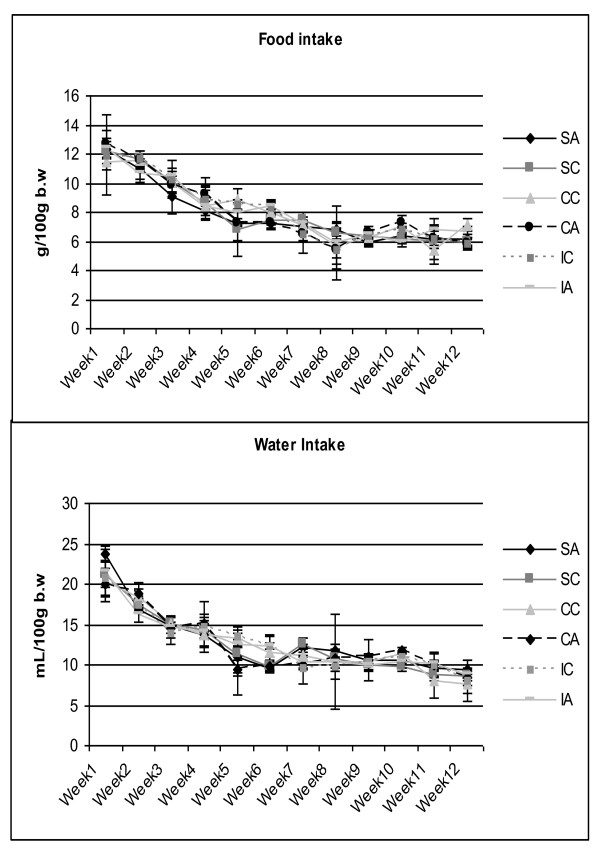
**Food intake and water intake of the animals at weaning (28 days) until the end of the experiment (120 days)**. Results are expressed as means ± standard deviation of 10 animals per group. SA: Sedentary alloxan, SC: Sedentary control, CC: Continuous training control, CA: Continuous training alloxan, IC: Intermittent training control and IA: Intermittent training alloxan.

In Figures 4A and 4B, the values related to food and water intake, respectively, are presented. No significant differences among the groups were observed in these parameters. Table 1 presents the data of the fasting and non-fasting (after glucose overload) serum glucose and insulin levels at 28 days of age. After glucose overload, the alloxan group presented higher serum glucose values than the control group.

**Table 1:** Fasting (12 hs) and non fasting (after glucose overload) serum glucose and insulin levels at weaning (28 days).

28 days	Alloxan	Control
Fasting serum glucose (mg/dL)	82.1 ± 8.6	81.8 ± 6.7
Non fasting serum glucose (mg/dL)	200.6 ± 35.0 *	155.3 ± 11.2
Fasting insulin level (ng/mL)	0.43 ± 0.27	0.61 ± 0.41
Non Fasting insulin level (ng/mL)	0.84 ± 0.39	0.79 ± 0.48

Results are expressed as means ± standard deviation of 10 animals per group. *, statistically significant different (student test, p < 0.05) from control group.

Table 2 shows the fasting and non-fasting (after glucose overload) serum glucose and insulin levels at 120 days of age. The IA group presented higher serum glucose values than the IC group after glucose overload.

**Table 2:** Fasting (12 hs) and non fasting (after glucose overload) serum glucose and insulin levels at 120 days.

120 days	SA	SC	CC	CA	IC	IA
Fasting serum glucose (mg/dL)	79.38 ± 6.62	78.93 ± 3.54	75.83 ± 4.28	77.61 ± 4.28	78.00 ± 7.05	77.74 ± 5.96
Non fasting serum glucose (mg/dL)	132.03 ± 16.38	115.74 ± 5.16	118.77 ± 8.07	124.42 ± 13.17	105.42 ± 5.41	125.44 ± 18.80^f^
Fasting insulin level (ng/mL)	0.84 ± 0.61	1.90 ± 1.04	2.10 ± 1.59	0.72 ± 0.44	1.28 ± 1.00	1.53 ± 0.94
Non fasting insulin level (ng/mL)	3.72 ± 2.71	2.74 ± 1.98	2.29 ± 1.66	1.97 ± 1.40	3.96 ± 2.55	3.71 ± 1.85

Results are expressed as means ± standard deviation of 10 animals per group. SA: Sedentary alloxan, SC: Sedentary control, CC: Continuous training control, CA: Continuous training alloxan, IC: Intermittent training control and IA: Intermittent training alloxan. Letter indicate a statistically significant difference among groups (Two- way ANOVA (3 × 2) and Bonferroni post hoc test, p < 0.05) referring to non-fasting serum glucose. f, statistically significant difference for IA vs. IC.

At 120 days of age it was evaluated the static insulin secretion by the pancreatic islets in response to different glucose concentrations (2.8 and 11.1 mM) which is presented in the table 3. It was observed significant differences among the groups. At concentration of 2.8 mM the SA group showed lower values when compared to corresponding control. The same was found in the glucose concentration 11.1 mM. Both exercise training protocols counteracted the alterations at 2.8 mM glucose concentration. Total serum protein and albumin levels, DNA content and the protein/DNA ratio in the gastrocnemius muscle are presented in Table 4. When total serum protein and albumin were analyzed, no differences among the groups were observed. In relation to the DNA content of the gastrocnemius muscle, the CA group showed higher values than the IA group. On the other hand, the protein/DNA ratio was higher in the IA group than in the CA group, which indicates higher muscle hypertrophy after intermittent training.

**Table 3:** Static insulin secretion (ng/5 islets × h) by the isolated pancreatic islets in response to different glucose concentrations (2

Glucose Concentration	SA	SC	CC	CA	IC	IA
2.8 mM	0.92 ± 0.17^a^	1.56 ± 0.40	1.52 ± 0.36	1.32 ± 0.44	0.63 ± 0.28^h, i^	1.97 ± 0.48^c, d, f^
11.1 mM	10.65 ± 3.76^a^	25.00 ± 6.08	26.36 ± 7.76	17.43 ± 3.13^e^	4.69 ± 2.28^h, i^	5.94 ± 2.60^d^

Results are expressed as means ± standard deviation of 10 animals per group. SA: Sedentary alloxan. SC: Sedentary control. CC: Continuous training control. CA: Continuous training alloxan. IC: Intermittent training control and IA: Intermittent training alloxan. Letter indicate a statistically significant difference among groups (Two- way ANOVA (3 × 2) and Bonferroni post hoc test. p < 0.05) referring to non fasting serum glucose. a. statistically significant difference for SA vs SC; c. statistically significant difference for SA vs IA; d. statistically significant difference for CA vs IA; e. statistically significant difference for CA vs CC; f. statistically significant difference for IA vs. IC; h. statistically significant difference for SC vs IC; i. statistically significant difference for CC vs IC.

**Table 4:** Total serum protein and albumin levels, protein level, DNA content and the protein/DNA ratio in the gastrocnemius muscle of the animals at 120 days.

	SA	SC	CC	CA	IC	IA
Total serum protein (g/L)	7.08 ± 0.08	7.05 ± 0.22	7.12 ± 0.16	7.20 ± 0.13	7.17 ± 0.18	7.19 ± 0.11
Serum albumin (g/L)	4.78 ± 0.37	4.82 ± 0.28	4.98 ± 0.36	4.87 ± 0.45	5.04 ± 0.36	5.33 ± 0.86
Protein gastrocnemius (mg/100 mg)	6.01 ± 1.37	6.09 ± 0.86	6.51 ± 1.25	5.39 ± 1.32	5.55 ± 1.22	6.38 ± 0.81
DNA gastrocnemius (mg/100 mg)	0.053 ± 0.013	0.058 ± 0.010	0.069 ± 0.016	0.071 ± 0.021	0.051 ± 0.012	0.050 ± 0.016^d^
Protein/DNA gastrocnemius	110.35 ± 20.37	108.02 ± 27.80	95.35 ± 15.17	84.95 ± 37.15	115.61 ± 39.29	146.27 ± 69.28^d^

Results are expressed as means ± standard deviation of 10 animals per group. SA: Sedentary alloxan, SC: Sedentary control, CC: Continuous training control, CA: Continuous training alloxan, IC: Intermittent training control and IA: Intermittent training alloxan. Letter indicate a statistically significant difference among groups (Two- way ANOVA (3 × 2) and Bonferroni post hoc test, p < 0.05) referring DNA and Protein/DNA ratio. d, statistically significant difference for CA vs. IA.

Protein synthesis and degradation in the soleus muscle did not differ among the groups (Table 5).

**Table 5:** Protein synthesis and degradation in the soleus muscle of the animals at 120 days.

	SA	SC	CC	CA	IC	IA
Protein synthesis (pmol/mg.h)	11.06 ± 2.21	12.77 ± 5.76	19.40 ± 3.82	13.68 ± 6.37	14.46 ± 2.59	11.14 ± 2.21
Protein degradation (pmol/mg.h)	350.56 ± 90.71	287.93 ± 37.38	271.29 ± 89.94	244.04 ± 44.26	387.00 ± 95.74	322.49 ± 93.99

Results are expressed as means ± standard deviation of 10 animals per group. SA: Sedentary alloxan, SC: Sedentary control, CC: Continuous training control, CA: Continuous training alloxan, IC: Intermittent training control and IA: Intermittent training alloxan.

## 4. Discussion

It is known that insulin has effects on glucose, fat and protein metabolism. Particularly in protein metabolism, insulin increases the intensity of amino acid transport across the cell membrane, which increases the available amino acids for protein synthesis. In diabetes mellitus, due to changes in insulin production and/or action, protein metabolism is altered. In turn, physical exercise promotes muscle protein anabolism, which results in morphological and metabolic adaptations in skeletal muscle.

Currently, it has been observed that high-intensity exercise can effectively increase muscle strength and mass and improve physical performance and functional capacity. Thus, the present study investigated the effects of intermittent and continuous training on protein metabolism in neonatal alloxan-administered rats.

In this study, the weight gain of animals was analyzed, and it was observed that the neonatal alloxan administration did not affect weight gain. Similar results were reported by Ribeiro et al. [24] using the same experimental model used in the present study.

Moreover, the sedentary control group showed higher gains in body weight than the control group that was subjected to the intermittent training protocol, which demonstrates a beneficial effect of this protocol on weight control. Furthermore, it is known that overweight or obesity are risk factors for the onset of T2DM [42]. Studies show that adipose tissue distribution is changed in type 2 diabetes mellitus [43]. Thus this study analyzed the adipose tissue in the retroperitoneal, subcutaneous and mesenteric regions. No difference was found in fat mass in the different regions when compared the groups that received the diabetogenic drug and controls. However, significant difference was found between the control animals after physical training, showing a smaller deposit of retroperitoneal fat in animals that performed continuous training compared to intermittent training. This demonstrates the beneficial effect of exercise to reduce abdominal fat [44] which high risk of developing metabolic complications such as impaired glucose tolerance, insulin resistance [45].

With respect to food and water intake of the animals, no difference was found among the groups, which shows that neonatal alloxan administration did not lead to polyphagia or polydipsia, which are usually observed in adult alloxan-mediated diabetic animals [46].

T2DM leads to glucose intolerance and insulin resistance, therefore, to evaluate changes in glucose homeostasis, the fasting and non-fasting (after glucose overload) serum glucose and insulin levels were determined at 28 and 120 days of age. At 28 days, glucose intolerance, which was indicated by the higher values of glycemia 30 min after the oral glucose overload, was observed to a greater extent in the alloxan group compared to the control group, which confirms the findings of a previous study [23]. At 120 days, glycemia after glucose overload was higher in the intermittent trained alloxan group compared to the corresponding control group, which was evidence for the differences in glucose homeostasis between the alloxan and control groups. After static insulin secretion by the isolated pancreatic islets in response to different glucose concentrations evaluation, it was evident the alloxan effect, because at lower and high glucose concentrations (2.8 and 11.1 mM) the sedentary alloxan animals had lower insulin secretion compared to the sedentary controls. On the other hand in the Oliveira et al. [22] study, using similar experimental model, no significant difference was found between animals that received the drug in 2 days of age and the control rats in the insulin secretion at different glucose concentrations (2.8 e 16.7 mM). Furthermore, it was observed in the present study that physical training has an important role in improving insulin secretion. At low glucose concentrations (2.8 mM) both exercise training protocols avoided the decrease in glucose induced insulin secretion caused by alloxan administration. Similar results were described in a previous study where the rats that received alloxan at 2 days of age and performed continuous training had better insulin secretory response compared to the sedentary animals [22].

The growth of organs can be caused by an increase in cell number (hyperplasia), an increase in cell size (hypertrophy) or by both processes simultaneously. The total cell number can be measured, with exceptions, by determining the total DNA content of the organ and dividing by a constant that represents the DNA content per diploid nucleus in the species being studied [38]. Determined the number of cells, the average weight, the protein content and the RNA content per cell, among others, can be assessed by determining the total quantity of each element and dividing by the number of cells. The result can be expressed as weight/DNA, protein/DNA, RNA/DNA ratios, etc. [38].

An increase in DNA content represents one type of growth: increased cell number; an increase in the weight/DNA, protein/DNA and RNA/DNA ratios represent another aspect of growth: increased tissue mass without an increase in cell number [38]. In the rats between 0 and 20 days old, all organs grow by cell division only, whereas between 21 and 42 days, most organs, including striated skeletal muscle, grow by both cellular hyperplasia and hypertrophy; between 64 and 86 days, all organs grow by cellular hyperplasia[38]. Physiological alterations during the hyperplastic and/or hypertrophic growth of an organ may result in changes in the number and/or size of the cells in the organ.

Therefore, in the present study, the protein, DNA content and protein/DNA ratio in the gastrocnemius muscle were analyzed. The alloxan group that was subjected to the continuous training showed higher DNA content and lower protein/DNA ratios than that of the intermittent training alloxan group, which indicates that continuous training was effective in promoting muscle hyperplasia, whereas intermittent training resulted in greater muscle hypertrophy. In previous studies, it was showed that exercise training increased serum and muscle IGF-1 concentrations in diabetic rats [47-50]. Thus, in the present study, the alterations in muscle mass in the alloxan groups after exercise training could be related to a possible increase in IGF-1 levels.

In an attempt to elucidate the possible mechanisms involved in cellular growth alterations in skeletal muscle that are induced by exercise training, protein synthesis and degradation rates were evaluated in the soleus muscle, but no differences were observed among the groups. Fedele et al. [28] also found no change in muscle protein synthesis in diabetic rats after acute resistance training. On the other hand, Farrell et al. [51] showed that diabetic rats presented a higher muscle protein synthesis after moderate endurance training.

Compared to aerobic training, which involves mainly oxidative fibers, generates increased mitochondrial activity and has a reduced effect on protein metabolism, high intensity training involves a greater participation of glycolytic type fibers and a greater increase in mass as well as increased protein synthesis. Therefore, in the present study, protein changes may not have been evident after intermittent training because the muscle fibers that were analyzed have predominantly oxidative characteristics.

In conclusion, neonatal alloxan administration reduced glucose-induced insulin secretion by pancreatic islet. Although the alloxan administered rats did not show overt diabetes mellitus, the glucose homeostasis was impaired, as indicated by their glucose intolerance. The continuous and intermittent training protocols were effective in preventing the reduction in glucose-induced insulin secretion by pancreatic islets of the alloxan administered animals. The continuous training also improved glucose tolerance. Both exercise raining protocols were effective in altering muscle growth by hyperplasia and hypertrophy, respectively, in the alloxan animals. Additional studies are needed to elucidate the possible mechanisms that are involved in these alterations.

## List of abbreviations

**T1DM**: Type 1 diabetes mellitus; **T2DM**: Type 2 diabetes mellitus; **WL**: total weekly training load.

## Competing interests

The authors declare that they have no competing interests.

## Authors' contributions

CR conceived the study, developed the study protocol, reviewed the references, collected and analyzed the data, and wrote the paper. LTC, RAD, MBA, ACG, LPM and GGA, JDB, participated in the design of the study, reviewed the manuscript, collected the data, and collaborated on the biochemical dosages. MARM conceived the study, participated in its design and coordination and helped in the drafting of the manuscript. All authors read and approved the submission of the final manuscript.
